# The effect of growth hormone supplementation in poor ovarian responders undergoing IVF or ICSI: a meta-analysis of randomized controlled trials

**DOI:** 10.1186/s12958-020-00632-w

**Published:** 2020-07-29

**Authors:** Peiwen Yang, Ruxing Wu, Hanwang Zhang

**Affiliations:** Reproductive Medicine Center, Tongji Hospital, Tongji Medical College, Huazhong University of Science and Technology, 1095 Jiefang Avenue, Wuhan, 430030 People’s Republic of China

**Keywords:** Growth hormone, Poor ovarian response, In vitro fertilization, Intracytoplasmic sperm injection

## Abstract

**Purpose:**

The aim of this meta-analysis was to evaluate the effect of growth hormone (GH) supplementation in poor responders undergoing in vitro fertilization (IVF) or intracytoplasmic sperm injection (ICSI).

**Methods:**

PubMed, MEDLINE and Cochrane Library databases were searched for the identification of relevant randomized controlled trials. Outcome measures were live birth rate, clinical pregnancy rate, miscarriage rate, cycle cancelation rate, number of retrieved oocytes and total dose of gonadotropin.

**Results:**

Fifteen randomized controlled trails (RCTs) involving 1448 patients were eligible for the analysis. GH supplementation improved live birth rate (RR, 1.74; 95% CI, 1.19–2.54), clinical pregnancy rate (RR, 1.65; 95% CI, 1.31–2.08) and retrieved oocytes number (SMD, 0.72; 95% CI, 0.28–1.16), while reducing cancelled cycles rate (RR, 0.62; 95% CI, 0.44–0.85) and dose of Gonadotropin (SMD,-1.05 95% CI, − 1.62 - -0.49) for poor ovarian response patients. Besides, there was no significant difference in the miscarriage rate between GH group and control group.

**Conclusions:**

Based on the limited available evidence, growth hormone supplementation seems to improve IVF/ICSI outcomes for poor ovarian responders. Further randomized controlled trials with large sample sizes are required to clarify the effect of GH adjuvant therapy in the treatment of women with poor ovarian response.

## Background

Poor ovarian response (POR) is a condition that in a group of IVF and ICSI cycles, despite the appropriate ovarian stimulation, the number of oocytes collected is below the expected value [1].

POR presents approximately in 5–18% in all assisted reproductive technology (ART) cycles, with a pregnancy rate as low as 2—4% [2]. Therefore, POR is considered as one of the success-limiting factors for IVF/ICSI outcomes [3]. The definition of POR has varied over time. In 2011, the European Society of Human Reproduction and Embryology (ESHRE) published the BOLOGNA criteria as a standardized definition. According to these criteria, poor responders are diagnosed with at least two of the three following criteria:1) advanced maternal age (≥40 years) or any other risk factor for POR, 2) a previously characterized POR cycle (≤3 oocytes with a conventional stimulation protocol), 3) an abnormal ovarian reserve test (i.e. antral follicle count < 5–7 follicles or AMH < 0.5–1.1 ng/mL) [4]. Various treatment including different stimulation protocols and adjuvant therapies have been applied to Poor ovarian responders; however, the management of poor ovarian responders is still a clinical challenge.

Growth hormone is a peptide hormone secreted primarily by pituitary gland, and participates in cell growth, development and metabolism [5]. Growth hormone receptors (GHRs) have been shown to be expressed in ovarian granulosa, theca cells, oocytes, cumulus cells, mammary glands, placenta and uterus [6]. It is reported that GH raises ovary sensitivity to follicule-stimulating hormone (FSH) [7], regulates ovarian function [8], promotes follicular maturation [9], enhances proliferation of the thecal and granulosa cells [10], and improves follicular development [11]. GH has been applied in the treatment of infertility, especially for poor responders. Some studies showed that GH administration during ovarian stimulation can improve IVF/ICSI outcomes, such as oocyte quality, pregnancy rate, and live birth [12–14]. In 2010, a Cochrane review suggested that due to few number of RCTs and small sample size, the role of GH in IVF/ICSI needs further research [15]. In 2017, a meta-analysis done by Li et al. reported that the GH addition can significantly improve the clinical pregnancy rate and live birth rate. Since then, five new RCTs studying the influence of GH addition on IVF/ICSI outcomes have been published [16–20].

Therefore, the aim of this meta-analysis is to screen and extract RCTs and evaluate the use of GH for POR patients undergoing IVF/ICSI.

## Methods

### Literature search strategy

Two independent authors (PWY and RXW) performed a systematically search of the PubMed, MEDLINE and Cochrane Library databases for literature published covering the period until February 2020. The search strategy was performed by consecutively entering the following Medical Subject Headings (MeSH) terms and free word to generate subsets of studies: i) ‘GH’ or ‘growth hormone’, ii) ‘Poor response’ or ‘low response’, ‘Diminished ovarian reserve’ or ‘Premature ovarian aging’. and iii) ‘Randomized controlled trial’ or ‘RCTs’. These subsets were combined together by ‘AND’. We also manually screened the reference lists of the retrieved articles to identify additional studies. No language limitations were applied.

### Eligibility criteria

The inclusion criteria were:

1. administration of growth hormone in the intervention;
2. inclusion of women characterized as poor responders;
3. inclusion of women undergoing IVF or ICSI, with any ovarian stimulation protocol;
4. the selected articles were RCTs;
5. a report of at least one of the following outcomes
6. Primary outcomes: live birth rate, clinical pregnancy rate
7. Secondary outcomes: miscarriage rate, cycle cancelation rate, number of retrieved oocytes, total dose of gonadotropin.

Secondary studies (i.e. systematic reviews, meta-analyses), and studies in which an additional drug was administered in conjunction with growth hormone were excluded.

### Data extraction and quality assessment

Two reviewers (PWY and RXW) selected the studies independently and extracted data for each study; any disagreement between the two reviewers responsible for data extraction was resolved by discussion. The extracted data included the following: first author, publication year, study design, inclusion and exclusion criteria, number of participants, interventions, controlled ovarian hyperstimulation (COH) protocols and outcomes (live birth rate, clinical pregnancy rate, miscarriage rate, cycle cancelation rate, number of oocyte retrieved, total dose of Gonadotropin). Articles were assessed for the risk of bias using the following parameters: random sequence generation, allocation concealment, blinding, incomplete outcome data, selective reporting and other bias.

### Statistical analysis

The Revman 5.3 software (Cochrane Collaboration, Copenhagen) was used for meta-analysis. Dichotomous results were analyzed by calculating the relative risks (RRs) with 95% confidence intervals (CIs). Continuous variables are expressed as the standardized mean differences (SMDs) with 95% CIs. Meta-analysis were performed using fixed and random effect models based on heterogeneity. The heterogeneity between studies was evaluated with Cochran’s Q and the I^2^ statistic, random effects model was performed if significant heterogeneity was identified between studies (*p* < 0.1, I^2^ > 50%). Otherwise, fixed effect model was applied. A funnel plot was used to evaluate publication bias.

## Results

**Fig. 1:**
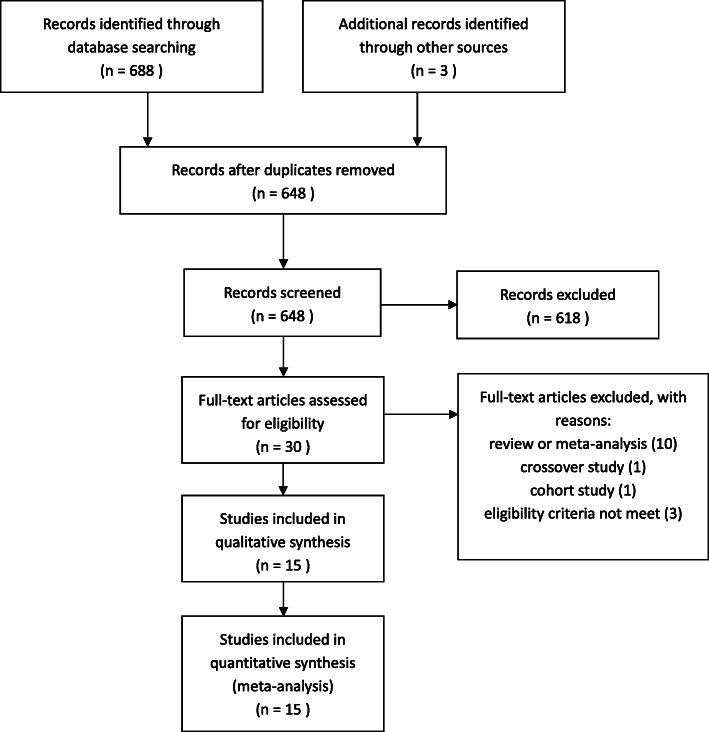
Flow Diagram

**Fig. 2:**
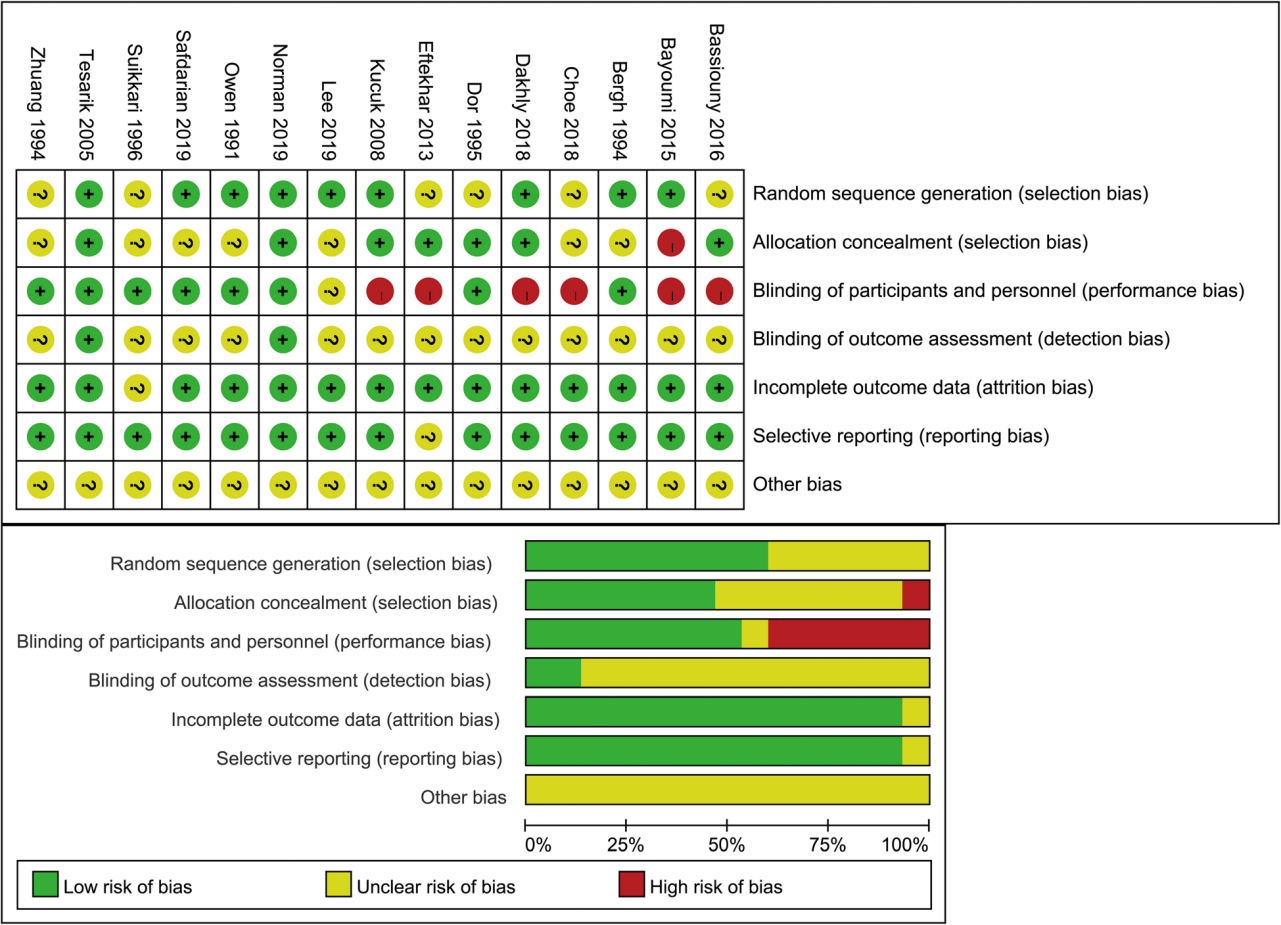
Quality control evaluations of included studies.? = unclear, + = low risk,–=high risk

The search strategy yielded 691 studies in total. Of these, we excluded 43 duplicates, after reading the titles and abstracts, 618 irrelevant articles were excluded. Full copies of the 30 remaining studies were retrieved. 15 articles were excluded for the reasons described in Fig. 1. Only 15 studies fulfilled the selection criteria. Finally, we included 15 RCTs [14, 16–29], with a total of 1448 patients, further details on these studies are listed in Table 1. All 15 studies were published between 1991 and 2019. All articles were intended to compare whether growth hormone could improve the IVF/ICSI outcomes of patients diagnosed with diminished ovarian reserve and/or poor ovarian response. The quality control evaluations of all studies are listed in Fig. 2.

**Table 1:** Characteristics of the studies included

Study	Intervention	GH	Control	Inclusion criteria	Stimulation protocol
Safdarian L (2019) [20]	GH 2.5 mg/day from day 8 or 0.1 mg/day from previous cycle day 3 ^f^	70	35	any two of three criteria to be met: age ≥ 40 years; oocytes≤3; AFC < 5–7 or AMH < 0.5–1.1 ng/ml	GnRH antagonist protocol
Norman RJ (2019) [19]	GH 12 IU/day from day 1 of stimulation ^a^	65	65	oocytes≤5; age ≤ 41; FSH ≤ 15 IU/l	GnRH antagonist protocol
Lee YX (2019) [18]	GH 4, 4, 2 IU for three successive days ^a^	94	90	age ≥ 40; oocytes≤3; AFC < 5–7 or AMH < 0.5–1.1 ng/ml	Long GnRH agonist protocol
Dakhly DMR (2018) [17]	GH 2.5 mg from previous cycle day 21 ^b^	120	120	any two of three criteria to be met: age ≥ 40 years; oocytes≤3; AFC < 5–7 or AMH < 0.5–1.1 ng/ml	Long GnRH agonist protocol
Choe SA (2018) [16]	GH 20 mg three times at mid-luteal, late luteal, and menstrual cycle day 2 ^c^	62	65	age ≥ 40; oocytes≤3; AFC < 5–7 or AMH < 0.5–1.1 ng/ml	GnRH antagonist protocol
Bassiouny YA (2016) [14]	GH 2.5 mg from day 6 of stimulation ^b^	68	73	any two of three criteria to be met: age ≥ 40 years; oocytes≤3; AFC < 5–7 or AMH < 0.5–1.1 ng/ml	GnRH antagonist protocol
Bayoumi YA (2015) [21]	GH 2.5 mg from day 6 of stimulation ^f^	84	88	any two of three criteria to be met: age ≥ 40 years; oocytes≤3; AFC < 5–7 or AMH < 0.5–1.1 ng/ml	microflare protoco
Eftekhar M (2013) [22]	GH 4 IU/day from previous cycle day 21 ^c^	40	42	failed IVF cycles≥1; oocytes≤3	GnRH antagonist protocol
Kucuk T (2008) [23]	GH 12 IU/day from previous cycle day 21 ^b^	31	30	responded poorly to high dose gonadotropin in first cycle	GnRH agonist long protocol
Tesarik J (2005) [24]	GH 8 IU/day from day 7 of stimulation ^a^	50	50	age 41–44 years	GnRH agonist long protocol
Suikkari A (1996) [25]	GH 4 IU/day or 12 IU/day from menstrual cycle day 3 ^d^	16	6	oocytes≤2 or ≥ 48 amples of hMG	GnRH-a flare up protocol
Dor J (1995) [26]	GH 18 IU on days 2, 4, 6 and 8 of the cycle ^e^	7	7	17-β oestradiol on hCG day< 501 pg/ml; follicles≤4; oocytes≤3	GnRH agonist short protocol
Bergh C (1994) [28]	GH 0.1 IU/kg during stimulation ^d^	9	9	failed IVF attempts≥2, oocytes< 5, age 25–38 years	GnRH agonist long protocol
Zhuang GL (1994) [27]	GH 12 IU on alternate days ^a^	12	15	previous sub-optimal response	GnRH agonist long protocol
Owen EJ (1991) [29]	GH 24 IU on alternate days during stimulation ^b^	13	12	oocytes≤6, embryos≤3	microflare protoco

^a^ Participants administrated with GH (Saizen, Merck Serono)

^b^ Participants administrated with GH (Norditropin, Novo Nordisk)

^c^ Participants administrated with GH (Eutropin, LG)

^d^ Participants administrated with GH (Genotropin, Kabi pharmacia)

^e^ Participants administrated with GH (Bio-Gropin; Bio-Technology General)

^f^ The product name of GH was not mentioned

### Live birth rate

**Fig. 3:**
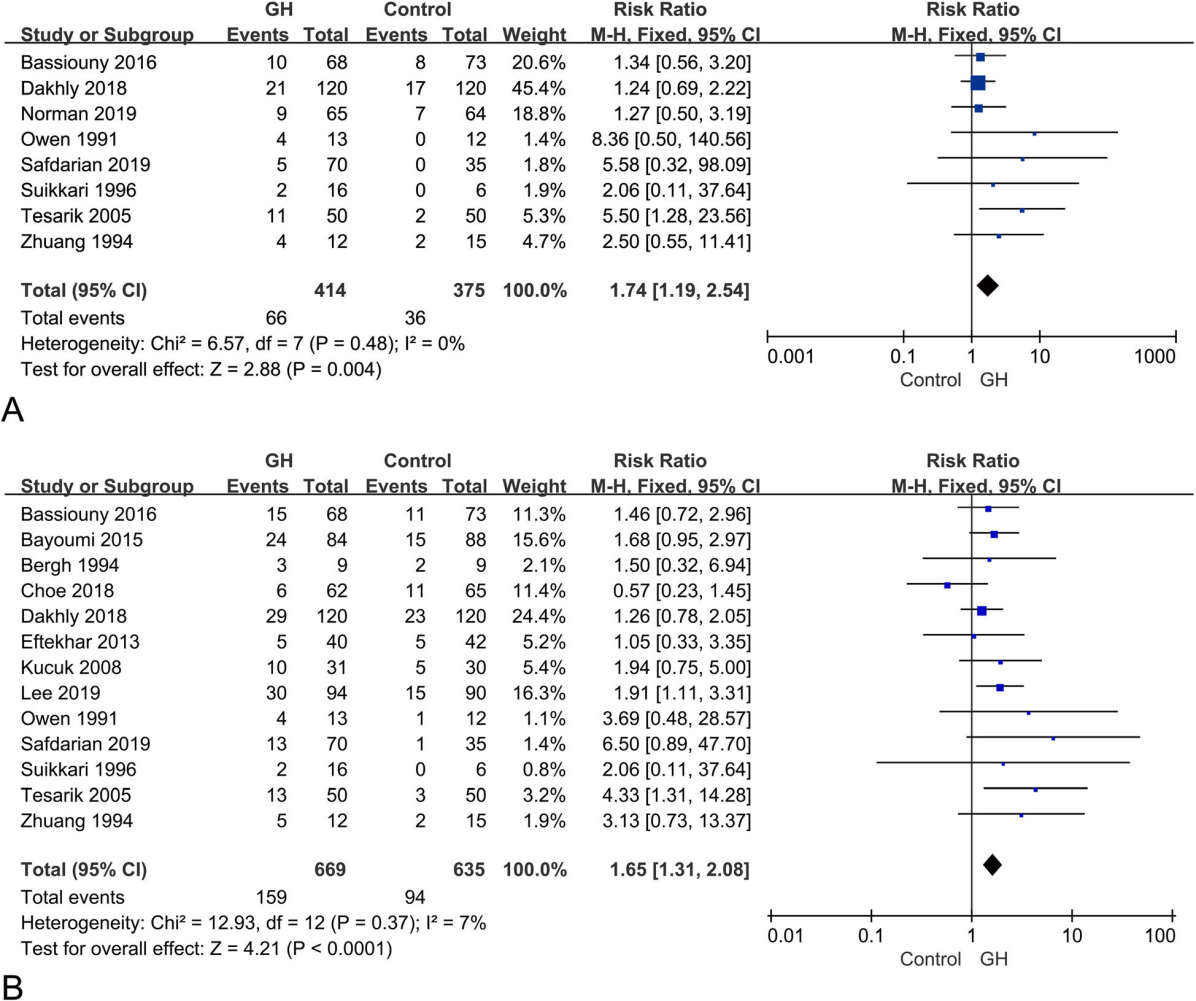
Forest plot for primary outcomes. **a** live birth rate; **b** clinical pregnancy rate

Eight studies [14, 17, 19, 20, 24, 25, 27, 29] including 789 patients (414 in the GH group and 375 in the control group) reported live birth rate. The meta-analysis indicated a statistically significant increase in the live birth rate in the GH group, compared to the control group (RR, 1.74; 95% CI, 1.19–2.54; *p* = 0.004), and no heterogeneity was observed between the studies (I^2^ = 0%; *p* = 0.48), as shown in Fig. 3a.

### Clinical pregnancy rate

Thirteen studies [14, 16–18, 20–25, 27–29] reported clinical pregnancy rate for 1304 patients (669 in the GH group and 635 in the control group). The meta-analysis (Fig. 3b) showed that GH addition could significantly increase the clinical pregnancy rate (RR, 1.65; 95% CI, 1.31–2.08; *p* < 0.0001). There was low heterogeneity between studies (I^2^ = 7%; *p* = 0.37).

### Miscarriage rate

**Fig. 4:**
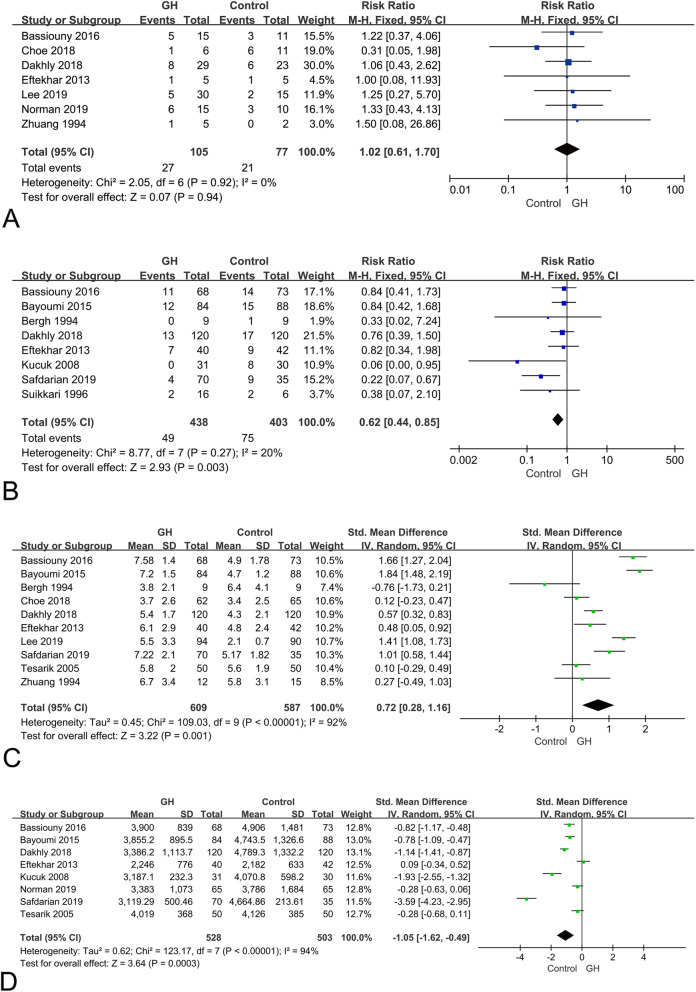
Forest plot for secondary outcomes. **a** miscarriage rate; **b** cycle cancelation rate; **c** number of retrieved oocytes; **d** total dose of gonadotropin

As shown in Fig. 4a, a total of 7 studies [14, 16–19, 22, 27] reported miscarriage rate. The meta-analysis indicated no significant difference in the miscarriage rates between the GH group and control group (RR, 1.02; 95% CI, 0.61–1.70; *p* = 0.94). No heterogeneity was observed between the studies (I^2^ = 0%; *p* = 0.92).

### Cycle cancellation rate

With respect to the cancellation rate of IVF/ICSI cycles, 8 studies [14, 17, 20–23, 25, 28] including 841 patients (438 in the GH group and 403 in the control group) were eligible. The meta-analysis (Fig. 4b) revealed that GH addition group had significant lower cancellation rate (RR, 0.62; 95% CI, 0.44–0.85; *p* = 0.003) compared to control group. The heterogeneity between studies was low (I^2^ = 20%; *p* = 0.27).

### Number of retrieved oocytes

A total of 13 studies reported number of retrieved oocytes, two of which [25, 26] lacked standard deviation of data, and one study [29] lacked the specific data of retrieved oocyte number. Finally, ten studies [14, 16–18, 20–22, 24, 27, 28] including 1196 patients (609 in the GH group and 587 in the control group) were eligible for the meta-analysis. As shown in Fig. 4c, the number of retrieved oocytes was significantly increased in GH group, compared to the control group (SMD, 0.72; 95% CI, 0.28–1.16; *p* = 0.001). However, significant heterogeneity was detected among these studies (I^2^ = 92%; *p* < 0.00001), therefore, the random effects model was performed.

### Total dose of gonadotropin

A total of 12 studies reported total dose of gonadotropin, three of which [25, 26, 28] lacked standard deviation of data, and one study [29] didn’t report the specific data of gonadotropin dosage. The meta-analysis finally included 8 studies [14, 17, 19–24] for 1031 patients (528 in the GH group and 503 in the control group). The result indicated a significant decrease in the gonadotropin dosage with the administration of GH (SMD,-1.05 95% CI, − 1.62 - -0.49; *p* = 0.0003). The random effects model was performed because of the high heterogeneity among these studies (I^2^ = 94%; *p* < 0.00001), as shown in Fig. 4d.

### Publication bias

**Fig. 5:**
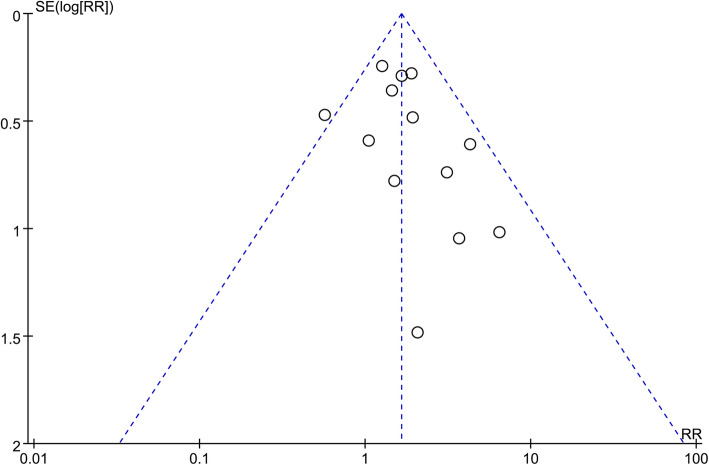
Funnel plot of the studies represented in the meta-analysis

Funnel plots were used to evaluate the potential publication bias. The funnel plot for the outcome of clinical pregnancy rate was asymmetric, as each point was scattered in Fig. 5. The potential publication bias could not be excluded.

## Discussion

The success of IVF/ICSI cycles is highly dependent on the number of retrieved oocytes that form qualified embryos for transfer. Low number of mature oocyte due to decreased ovarian reserve is a success-limiting factor for IVF/ICSI cycle outcomes [30]. Improving the IVF/ICSI outcomes is a challenge to infertility experts. Various efforts have been tried for improving IVF/ICSI outcomes of POR patients, including growth hormone administration. However, the effect of GH on POR patients has not been precisely determined yet.

Therefore, this meta-analysis was performed to provide a more precise estimate of the effect of GH supplementation for POR patients undergoing IVF/ICSI. Our meta-analysis included fifteen RCTs, and the results indicated that GH supplementation is associated with higher live birth rate in IVF/ICSI patients with POR. More oocytes were retrieved in the GH group for POR women in the IVF/ICSI cycle. GH supplementation might improve clinical pregnancy rate in our study, however, the potential publication bias for this outcome could not be excluded. Besides, the cancellation rate and gonadotropin dosage were decreased with the administration of GH, and no differences were observed in the miscarriage rate between the GH and control groups.

A previous meta-analysis done by Li et al. in 2017 [31] suggested that GH application significantly improved clinical pregnancy rate, live birth rate, collected oocytes number, meanwhile decreased cancelled cycles rate and dose of gonadotropin. Comparing to this study, five new RCTs [16–20, 26, 27] were included in our meta-analysis. The results of the two studies were similar. Additionally, our meta-analysis reported no significant difference in miscarriage rate between the GH and control groups, and Li et al. demonstrated more MII oocyte number, higher E_2_ on HCG day, no influence on implantation rate and fertilization rate with GH administration. Another meta-analysis [32] also indicated that GH supplementation improved clinical pregnancy rate and live birth rate. In a recent systematic review and network meta-analysis [33] comparing the effectiveness of various adjuvant treatment options to POR patients, GH treatment was reported to resulting in higher number of oocyte retrieved and lower dosages of gonadotropins for ovarian stimulation. These results were similar to our study. The Cochrane review published in 2010 [15] also demonstrated a statistically significant difference in both live birth rates and pregnancy rates favouring the use of adjuvant growth hormone in IVF protocols in POR women, however, the Cochrane review showed no significant difference in collected oocytes number with GH supplementation, which was different from our study. And a meta-analysis including 6 randomized controlled trials and 5 controlled clinical trials [34] reported that the clinical pregnancy rates between GH group and control group were similar in POR women in IVF/ICSI cycles, the difference may be associated with different included article type or different analysis methods.

The present meta-analysis demonstrates that GH supplementation in IVF protocol was an adjuvant treatment benefit to the IVF outcomes in patients with POR, the detailed mechanisms of which is still being investigated. GH can attach growth hormone receptors on oocytes, thus influencing their function. Previous studies have shown that GH supplementation might promote nuclear maturation of denuded human oocytes [35–37], improve oocyte quality by stimulating the growth and function of granulose cells [38, 39], improve reactivity of ovary [40] and endometrial receptivity [5], which might contribute to better IVF outcome.

Our meta-analysis included numbers of prospectively designed RCTs, and involved a large amount of patients, which improves the statistical power. However, the meta-analysis has several limitations. First of all, although there was low heterogeneity in the analyses of primary outcomes, the heterogeneity between the studies was found on number of retrieved oocytes and total dose of gonadotropin, the sources of heterogeneity between the studies may be related to the different definition of POR, stimulation protocol, and GH treatment method. Secondly, some included studies had relatively small sample sizes. This may have influenced the validity and reliability of our conclusions. Finally, not all included RCTs had strict methods of randomization, blinding, allocation concealment, missing data treatment, studies chosen did not have data for all outcomes or lacked data for some outcomes, and the potential publication bias for outcome clinical pregnancy rate could not be excluded in the study, which may affect the conclusions.

Data from this meta-analysis provides support for GH supplementation in IVF/ICSI cycles. GH leaded to higher live birth rate, since live birth is the ultimate end-point and primary outcome of infertility interventions, it might be a main benefit to POR women. Adjuvant treatment with GH decreased cycle cancellation rate, which helps shorten the treatment time of IVF. GH supplementation did not increase miscarriage rate. Moreover, no sever adverse event was reported in the RCTs included in our meta-analysis except that Norman reported one trisomy 21 and one patent ductus arteriosus in GH group [19], and the Cochrane review [15] indicated that the use of growth hormone adjuvant in IVF did not increase adverse events in women who are considered poor responders. Therefore the GH adjuvant treatment seems to be beneficial and relatively safe. However, there has been no standard protocol regarding GH addition time and dosage so far. Studies have shown that GH may contribute to insulin resistance and may be in relation with cancer [39]. In view of this, further studies are needed to establish the threshold dosage and the administration protocol of GH.

## Conclusion

In conclusion, the results of this meta-analysis suggested that GH supplementation might improve live birth rate, clinical pregnancy rate and retrieved oocytes number for poor ovarian responders who are undergoing IVF/ ICSI. Besides, GH decreased cancelled cycles rate and dose of Gonadotropin for POR women, meanwhile having no influence in the miscarriage rate. However, further large-scale RCTs should be performed to determine the utility of GH adjuvant therapy in the treatment of women with poor ovarian response.

## Supplementary information

**Additional file 1.**

## Data Availability

Please contact author for data requests.
